# May I check your cap?

**DOI:** 10.7554/eLife.15570

**Published:** 2016-04-06

**Authors:** Elisabeth A Geyer, Shreoshi Majumdar, Luke M Rice

**Affiliations:** 1 Departments of Biophysics and Biochemistry, UT Southwestern Medical Center, Dallas, United States; 1 Departments of Biophysics and Biochemistry, UT Southwestern Medical Center, Dallas, United States; 1 Departments of Biophysics and Biochemistry, UT Southwestern Medical Center, Dallas, United StatesLuke.Rice@UTSouthwestern.edu

**Keywords:** microtubule dynamic instability, end tracking, microfluidics, TIRF microscopy, EB proteins, microtubules, None

## Abstract

Modernizing a classic technique to study microtubules has revealed that the stability of a microtubule is related to its growth rate.

**Related research article** Duellberg C, Cade N, Holmes D, Surrey T. 2016. The size of the EB cap determines instantaneous microtubule stability. *eLife* **5**:e13470. doi: 10.7554/eLife.13470

**Image** A new method can follow the fate of microtubule ends in higher detail than before

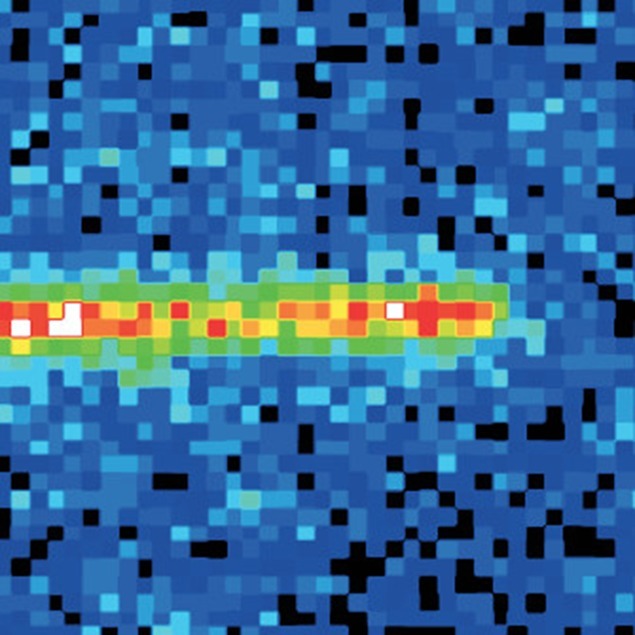

Microtubules are hollow cylindrical polymers that have important roles in chromosome segregation, organelle transport and other processes inside cells. Microtubules are built from protein subunits called αβ-tubulin and abruptly switch between growing and shrinking. This switching property is known as "dynamic instability", and has captivated scientists since it was discovered over 30 years ago (Horio and Hotani, 1986; Mitchison and Kirschner, 1984).

The switch from the growing state to the shrinking state is known as catastrophe, and is essential for microtubules to work correctly. Catastrophe occurs when the microtubule loses its stabilizing cap, which is a biochemically distinct region near the growing end. Despite substantial efforts, both the size of this stabilizing cap and its relationship to microtubule growth rates have remained obscure. Now, in eLife, Thomas Surrey and co-workers at the Francis Crick Institute and the London Centre for Nanotechnology – including Christian Duellberg as first author – use state-of-the-art methods to resolve these longstanding conundrums (Figure 1; Duellberg et al., 2016).

**Figure 1.**
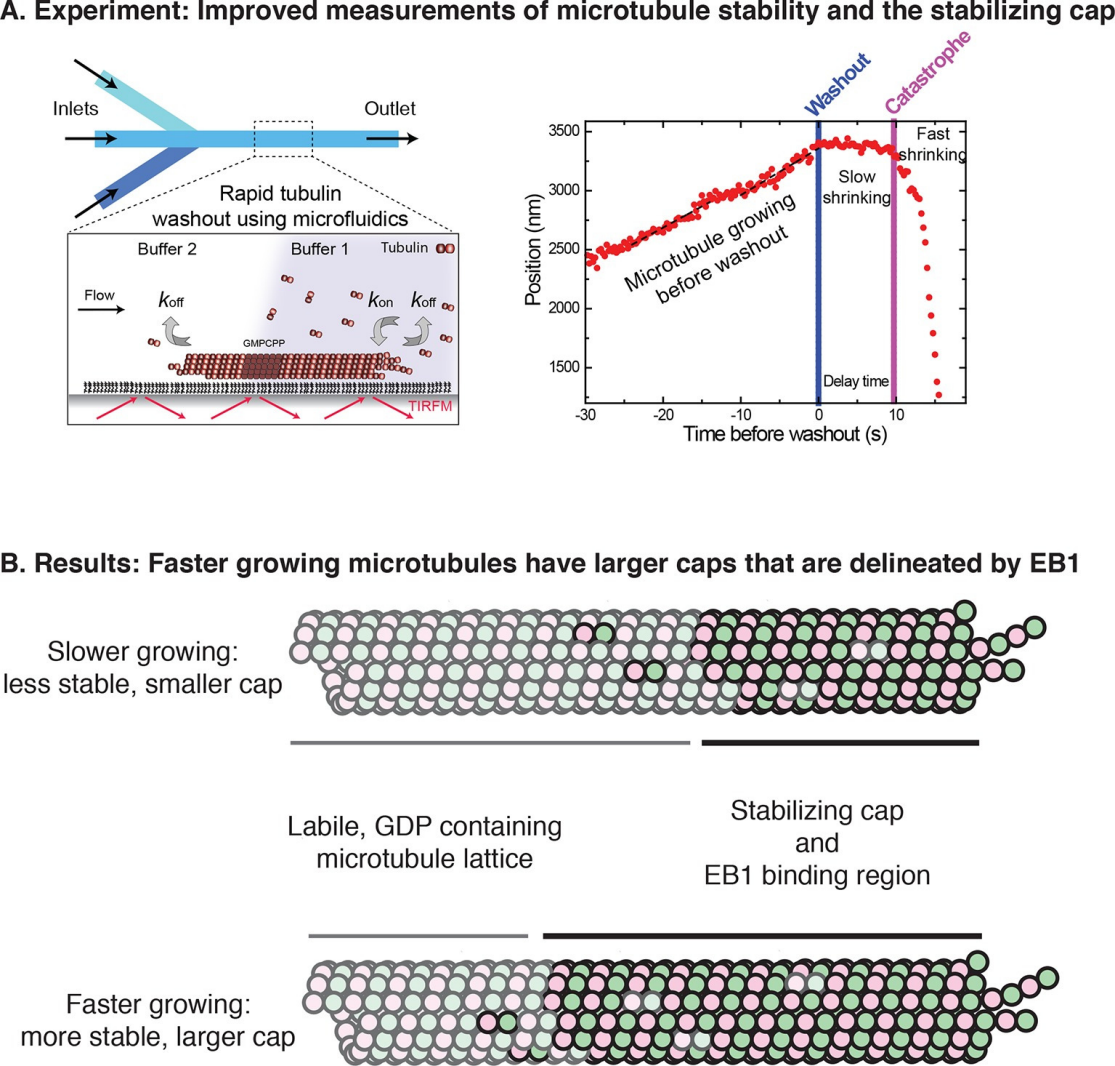
A modernized form of a classic technique enables the growth and stabilization of microtubules to be studied. (**A**) Left: Duellberg et al. used microfluidics to abruptly stop microtubule growth via the "washout" approach. Right: Sample data showing microtubule length versus time. Before washout, the microtubule grows steadily; after washout, it shrinks slowly for a time; and after catastrophe, it shrinks rapidly (Panel adapted from Figures 1A and 2A, Duellberg et al.). (**B**) Duellberg et al. observed correlations between the microtubule growth rate and the size of the stabilizing cap, which consists of GTP-bound αβ-tubulin subunits (indicated by the non-faded circles). The caps are marked by EB1 proteins (not shown explicitly).

In a simple sense, microtubule catastrophe results from a race between two competing processes. Microtubules grow by capturing αβ-tubulin subunits that are bound to a molecule called GTP. However, shortly after a new subunit is added to the polymer, its GTP molecule is hydrolyzed: this destabilizes the polymer and provides the driving force for catastrophe.

The time interval between the addition of the subunit and the hydrolysis of the GTP should create a cap that protects the growing microtubule against catastrophe. This simple view predicts that faster growing microtubules should have larger caps. Over the years, however, experiments to test this prediction have yielded conflicting results (Caplow and Shanks, 1996; Schek et al., 2007), resulting in a proliferation of different models that attempt to describe microtubule stabilization (reviewed in Bowne-Anderson et al., 2013).

“Washout” experiments are a classic way to measure microtubule stability and estimate cap size by suddenly diluting the solution around growing microtubules to stop their growth (Walker et al., 1991). After washout, the microtubule shrinks slowly for a short period of time before it begins to shrink rapidly. The time delay before rapid shrinking occurs is related to the size of the stabilizing cap. Using this approach, a classic early paper failed to find a relationship between the rate at which microtubules grow and their stability (Walker et al., 1991).

Duellberg et al. have now revitalized this washout approach by developing a new microfluidics-based method that enables much faster dilution (Figure 1A). This method also incorporates high-precision microtubule end tracking and averaging techniques previously developed by Surrey and co-workers (Maurer et al., 2014).

The new approach allowed Duellberg et al. to demonstrate that microtubules that are growing faster at the time of dilution experience a longer delay before they begin to rapidly shrink (Figure 1B). The cap size could also be estimated from the length of the slow-shrinking phase. Thus, microtubule growth rate and microtubule stability are correlated.

To provide more direct insight into the size of the cap and how it is lost, Duellberg et al. turned to the EB1 family of microtubule regulatory proteins. These proteins form ‘comets’ by binding to an extended region near the microtubule end (Bieling et al., 2007), and are thought to recognize unique structural features of the stabilizing cap (Zhang et al., 2015).

To determine whether microtubule stability is related to the size of EB comets, Duellberg et al. simultaneously monitored microtubule ends and fluorescently tagged EB proteins. They observed that faster growing microtubules recruited more EB protein at the growing end (as shown by more intense fluorescent ‘comets’). The number of high-affinity EB binding sites decreased exponentially after washout, and rapid shrinking began when the density of EB binding sites was reduced to about 20% of its maximal value.

Consistent with these findings, microtubules with more EB binding sites (brighter comets) at the time of dilution exhibited a longer time delay before rapid shrinking began. This time delay can also be predicted if the rates of three processes are known: microtubule growth, slow shrinkage and EB binding site loss.

By demonstrating that EB proteins label the cap region and therefore provide a way to visualize it, and by resolving a longstanding ambiguity about the relationship between the cap and microtubule growth rate, Duellberg et al. advance our understanding of microtubule stabilization. However, we cannot currently explain Duellberg et al.’s findings in terms of the conformations and biochemical properties of individual αβ-tubulin subunits. More generally, microtubules normally undergo catastrophe without the sudden dilution used by Duellberg et al.: defining the mechanisms that normally trigger catastrophe therefore remains another important challenge.

The high-quality data that Duellberg et al. report set a new standard for studies of other microtubule regulatory proteins. Their pioneering quantitative analyses will undoubtedly contribute to future advances in the understanding of dynamic instability and its regulation.
